# The Clinical Application of Anterolateral Thigh Flap

**DOI:** 10.1155/2011/127353

**Published:** 2011-11-28

**Authors:** Yao-Chou Lee, Haw-Yen Chiu, Shyh-Jou Shieh

**Affiliations:** Division of Plastic and Reconstructive Surgery, Department of Surgery, National Cheng Kung University Medical College and Hospital, Tainan 70428, Taiwan

## Abstract

The anterolateral thigh flap can provide a large skin paddle nourished by a long and large-caliber pedicle and can be harvested by two-team work. Most importantly, the donor-site morbidity is minimal. However, the anatomic variations decreased its popularity. By adapting free-style flap concepts, such as preoperative mapping of the perforators and being familiar with retrograde perforator dissection, this disadvantage had been overcome gradually. Furthermore, several modifications widen its clinical applications: the fascia lata can be included for sling or tendon reconstruction, the bulkiness could be created by including vastus lateralis muscle or deepithelization of skin flap, the pliability could be increased by suprafascial dissection or primary thinning, the pedicle length could be lengthening by proximally eccentric placement of the perforator, and so forth. Combined with these technical and conceptual advancements, the anterolateral thigh flap has become the workhorse flap for soft-tissue reconstructions from head to toe.

## 1. Introduction

Since Song et al. [1] introduced the anterolateral thigh flap in 1984, it gains popularity because of several advantages [2, 3]. First, the flap can be harvested simultaneously as two-team work. The operation time could be shortened. Second, the pedicle length is long enough to anastomosis with recipient vessels. The vein graft could be avoided. Third, the large caliber of pedicle vessels makes microanastomosis easier. Fourth, the flap could serve as fasciocutaneous, adipofascial, or myocutaneous flap as needed. Fifth, the flap can have great volume variability. Pliability could be achieved by primary thinning. Bulkiness could be added by incorporation of the deepithelialized skin or a portion of muscle cuff. Sixth, the lateral femoral cutaneous nerve can be included to provide as a sensate flap. Seventh, the flap pedicle could bridge the vascular gap as flow-through flap, especially in mangled extremities. Eighth, the donor site morbidity is minimal.

## 2. Flap Anatomy

### 2.1. Perforator

Both septocutaneous and musculocutaneous perforators were identified in the anterolateral thigh flaps. Initially, it was thought that septocutaneous route composes the dominance [1, 4]. Recently, the anatomic studies suggested that musculocutaneous route takes the majority [2, 5–7]. In Shieh et al.'s and Wei et al.'s reports, 83.2% and 87.1% of perforators were found to be musculocutaneous, respectively [2, 3]. The differences between each studies might relate to the bias of the selection of the perforators by different authors. Originally, the locations of the perforators in relation to the anterolateral thigh skin were described within 3 cm radius circle centered on the midpoint between the anterior superior iliac spine (ASIS) and superolateral corner of patella in most circumstances [5]. These perforators near the midpoint in the line linking the ASIS and superolateral part of patella were further classified as B (middle) perforators in Yu's study [8]. He also defined A (proximal) and C (distal) perforators based on 72 anterolateral thigh flaps' exploration. In his study, A perforators were presented in 49% of cases with almost equal distribution of septocutaneous and musculocutaneous perforators (52% versus 48%), while B and C perforators were presented in 93% and 63% of cases, with musculocutaneous dominance in 84% and 95%, respectively. In general, the proximal perforators have larger chance to be septocutaneous type, and the distal perforators usually are musculocutaneous in majority [8]. In 0.9–5.4% of cases, no perforators were found despite thorough examination [3, 7, 8].

### 2.2. Pedicle

The pedicle of the anterolateral thigh flap was believed to be the descending branch of lateral circumflex femoral artery (LCFA) which originated from profunda femoris artery [1, 3]. However, some skin perforators actually originate from elsewhere, such as transverse branch of LCFA, profunda femoris artery, or even trunk of femoral artery [2, 7, 8]. In Shieh et al.'s study [2], all flaps could be harvested successfully based on the perforators originated from descending branch of LCFA in 67.6% of cases and transverse branch of LCFA in 32.4%. In Kimata et al.'s [7] and Yu's [8] studies, the perforators arose from profunda femoris artery or trunk of femoral artery in 1.4–4% of cases. 

## 3. Clinical Considerations in Flap Design

### 3.1. Pedicle Length

The pedicle length is basically determined by the most proximal chosen perforator if more than one perforator is selected. In Yu's study, the mean pedicle length was 9.7 cm or 13.2 cm based on A or B perforator, respectively [8]. They did not measure the pedicle length if C perforator was used as the most proximal chosen one. In general, the average pedicle length was about 12 cm and as long as 20 cm could be achieved [2, 7]. If long pedicle length is required in clinical scenario, distal perforators should be used while size is reliable, or additional length could be obtained from proximal eccentric placement of the selected perforator in flap design.

### 3.2. Recipient Vessels

In the scalp, skull base, and upper face reconstruction, the choice of recipient vessel is superficial temporal artery and its concomitant veins. In the midface, lower face, and neck reconstruction, the choices of recipient arteries will be facial artery, superior thyroid artery, and transverse cervical artery. The recipient veins common used are the concomitant veins of the recipient arteries, external jugular vein, or internal jugular vein. In breast reconstruction, the recipient vessels could be the internal mammary artery and thoracodorsal artery. In abdominal wall reconstruction, the anterolateral thigh flap could be designed as proximal pedicled flap. In the upper abdomen where the rotational arc cannot reach pedicled flap, free flap with microanastomosis to the deep inferior epigastric vessels is suggested. From lower abdomen to knee, the pedicled flap could have a wide range of rotational arc based on proximally or distally pedicled design. For reconstruction of leg and foot, the free flap could be anastomosis to the either posterior tibia system or anterior tibia system in an end-to-side fashion to avoid disturbing the distal limb circulation.

### 3.3. Flap Components versus Defect Requirement

The anterolateral thigh flap takes the advantage to have different tissue components including skin, fat, fascia, and muscle. Therefore, they could be designed as fasciocutaneous, adipofascial, or myocutaneous flaps. In head and neck region, if dura or fascia sling is necessary to reconstruct the dura defect or oral competence, the fascia lata could be included in the flaps. If facial soft tissue augmentation is the goal, adipofascial flap could be designed. If another bone flap is necessary for bony reconstruction, the anterolateral thigh flap could be designed for skin coverage, and its distal pedicle end or its braches off the main pedicle, such as rectus femoris branch and vastus intermedius branch, could be served as recipient vessels in a “piggy-back” serial connection or parallel connection, respectively [9]. When the bulkiness is necessary for dead space obliteration, the flap could be harvested as myocutaneous flap to include part of vastus lateralis muscle or harvest more subdermal fat pad either by undermining or deepithelization. Sometimes, the flap was purposely used to treat infection, such as osteomyelitis; myocutaneous flap should be the first choices based on the reason that muscle could achieve better infection control [10]. When the flap's pliability is the factor what we concern, it could be primary thinning to use in the upper extremities, ankle, and foot skin coverage. If tendon reconstruction is needed for extremity tendon defect, the fascia lata could be used as tendon substitutes.

## 4. Clinical Applications

### 4.1. Head and Neck

#### 4.1.1. Scalp and Calvarias

Free tissue transfer for scalp and calvarial defects after tumor extirpation, trauma, or infection was reported to achieve satisfactory results [11–14]. The anterolateral thigh flap had the advantages to be harvested as adipofascial, fasciocutaneous, or myocutaneous flap according to the defect requirements. In the skull base defect, the fascia could be used for duraplasty. If dead space was present, the myocutaneous design could be helpful. However, the potential disadvantages of using the ALT flap for this type of defects included: (1) in many patients, it is too bulky even without muscle; (2) if used as a perforator flap without muscle, the perforators are more prone to compression (against the bone). Including a cuff of muscle around the perforators may prevent compression or kinking. If the flap is too bulky, using vastus lateralis muscle only with skin graft is another alternative.

#### 4.1.2. Face/Oral Cavity

The anterolateral thigh flap was most famous by its usage after oral cancer ablation. When the defect is limited in intraoral lining, the flap should be harvested for its maximal pliability. That could be done under the suprafascial dissection technique or primary thinning. When the defects are through and through, not only intraoral lining but also external skin coverage, the flap should be designed to have two skin paddles. When there is more than one perforator, the two skin paddles could be separated based on its own perforator. If only one perforator is identified in the flap harvesting, the two skin paddles could be bridged by deepithelizing the skin in between. When the defect involves the lip causing oral incompetence, the fascia lata could be used as static slings or dynamic slings to restore oral competence and to decrease saliva drooling [15]. The defect after hemiglossectomy needs to be reconstructed by a pliable flap to avoid disturbing the residual tongue motility. In the advanced tongue cancer while subtotal or total glossectomy will be conducted for tumor excision, the bulkiness should be recreated to prevent saliva pooling and to improve neotongue-palate contact while swallowing [16]. When the defect involves the maxillectomy, dead space could be tamponaded by muscle bulk or by using the part of the deepithelized flap. When the bone reconstruction is planned, the anterolateral thigh flap could provide outer skin coverage. Furthermore, its distal pedicle stump or its branches off the main pedicle could be used to hook up a second osseous flap. The anterolateral thigh could also be harvested in adipofascial fashion and served for facial augmentation [17].

#### 4.1.3. Pharyngoesophageal Reconstruction

Pharyngoesophageal reconstruction by anterolateral thigh fasciocutaneous flap gained great success in Yu et al.'s work [18]. According to their recent 114 patients' experience, excellent clinical and functional outcomes with minimal donor site morbidity and quick recovery could be expected by using an anterolateral thigh flap. Oral diet without tube feeding could be achieved in 91% of patients. Fluent speech could be achieved in 41% of patients with a primary tracheoesophageal puncture and 81% of patients with a secondary tracheoesophageal puncture. Fistulas and strictures only occurred in 9% and 6% of patients, respectively. The flap was designed to have a width of 9.4 cm to achieve a 3 cm diameter skin tube for neopharynx. For nearcircumferential defect (<2 cm wide strip of mucosa left), flap width was designed by subtracting the width of the remaining pharyngeal mucosa from 9.4 cm. An additional width of fascia was harvested to reinforce the skin tube suture lines. At the distal esophageal anastomosis, the anterior cervical esophageal end was incised longitudinally for 1.5 cm to prevent stricture. Whenever possible, two perforators should be included to create two skin paddles, one for the skin tube and the other for neck skin coverage and/or monitoring.

### 4.2. Trunk

#### 4.2.1. Postmastectomy Reconstruction

Anterolateral thigh flap could be an alternative choice for postmastectomy breast reconstruction if lower abdominal wall tissue transfer is contraindicated due to inadequate soft tissue volume, previous abdominoplasty, lower paramedian or multiple abdominal scars, and plans for pregnancy [19, 20]. Although gluteal flap was traditionally thought to be the second choice, the anterolateral thigh flap takes advantages of longer pedicle without the need of vein graft, superior quality of skin and fat, and two-team works without position change. When large tissue amount is necessary or in thin patients, anterolateral thigh flap could be harvested by extended subdermal undermining to include more adipose tissue.

#### 4.2.2. Chest Wall Reconstruction

In patients who suffered from chest wall malignancy necessitating extensive ablative surgery or patients who received radiotherapy complicated by radionecrosis, local or regional muscle flaps usually are violated and therefore limited their use in chest wall reconstruction. Free tissue transfer will be preferred by its superior vascularity. Anterolateral thigh flap is suggested by the advantages of long pedicle allowing extrathoracic vascular anastomosis, reliable skin flap for skin coverage, and being able to carry vastus lateralis muscle to improve local circulation and obliterate dead space. Extrathoracic vascular anastomosis is recommended by its away from previous injured recipient vessels in chest region, adequate vessels caliber with the anterolateral thigh flap pedicle, and minimal disturbance from the breathing moving while microanastomosis [21]. 

#### 4.2.3. Abdominal Wall and Pelvic Defect

The pedicled anterolateral thigh flap had wide arc for abdominal wall and pelvic reconstruction and was reported to be able to reach 8 cm above the umbilicus [22]. When the rotational arc could not reach the defect, free anterolateral thigh flap will be indicated. The choices of recipient vessels include extraperitoneal vessels such as inferior epigastric vessels, deep circumflex iliac vessels, superior epigastric vessels, and internal mammary vessels; intraperitoneal vessels such as gastroepiploic and jejunal vessels. When large defect is necessary, anterolateral thigh flap combined adjacent tensor fascia lata flap could be adopted. When the defects involve the abdominal fascia, vascularized fascia lata could be included in the design of anterolateral thigh flap to minimize the risk of hernia and avoid problems associated with synthetic materials [23]. Composite harvest with vastus lateralis muscle is useful for dead space filling after pelvic exenteration. Satisfactory results with minimal donor site morbidities could be achieved in such composite abdominal wall reconstruction [23, 24].

### 4.3. Extremities

Free flap reconstruction for mangled extremities salvage is indicated when major vessels, major nerves, tendons, or bones are exposed, especially when the local tissues are violated making local flaps infeasible [25]. The anterolateral thigh flap has several advantages in mutilated extremities reconstruction. First, the long and large vascular pedicle could be designed as flow-through pattern to reconstruct the major vessel's defect. Second, the vascularized fascia lata inclusion serves as a gliding plane for transferred or repaired tendons. Third, a large skin flap is reliable for vital structures coverage. Fourth, two-team approach and no need for position change can effectively decrease operation time.

Pedicled anterolateral thigh flap is able to reconstruct the extremity between the groin and knee either by proximally based or distally based. When distally based anterolateral thigh flap will be conducted, retrograde flow from lateral superior genicular artery or profunda femoral artery should be safely maintained by careful dissection within 10 cm above the knee and including a 0.5 cm cuff of vastus lateralis muscle with the intramuscular pedicle. Rotation arc could reach the upper third of leg based on the pivot point 3 to 10 cm above of the knee and an average 15 cm pedicle length [26, 27]. Shieh and Jou [28] transposed a proximal-based pedicled vastus lateralis muscle flap effectively treating chronic intractable hip infection. The flap filled the hip cavity with well-vascularized soft tissue able to resist infection and resulted in successful secondary total hip arthroplasty. It offers an ideal alternative procedure for dealing with this type of intractable hip infection. At the distal lower extremities, anterolateral thigh flap could be used as free tissue transfer by microanastomosis to the more superficial-seated recipient vessels, such as posterior tibia artery or dorsalis pedis artery. In further, the anterolateral thigh flap could be tailored to reconstructed patella or Achilles tendon defects by including the vascularized fascia lata [29–31]. This vascularized tendon substitute not only fastens healing but also is more resistant to infection. Good functional outcomes could be expected.

## 5. Summary

The anterolateral thigh flap has several advantages and great versatility for soft tissue reconstructions. However, its anatomic variations in perforator route and pedicle origin may impede the flap usage. By adapting the free-style concepts postulated by Wei et al. [32–34], the anterolateral thigh flap might be raised in a safer manner. The main principles of free-style flaps are mapping the skin vessels by hand-held Doppler device and being familiar with retrograde intramuscular perforator dissection. At first, the anterolateral thigh flap could serve as a model for practicing such techniques of free-style flaps. And finally, the anterolateral thigh flap can be reliably elevated with minimal donor-site morbidity for head-to-toe reconstruction based on these free-style harvesting techniques.
